# Cognitive enhancement through action video game training: great expectations require greater evidence

**DOI:** 10.3389/fpsyg.2014.00136

**Published:** 2014-02-19

**Authors:** Joseph Bisoglio, Timothy I. Michaels, Joshua E. Mervis, Brandon K. Ashinoff

**Affiliations:** ^1^Columbia University Medical Center, New YorkNY, USA; ^2^Manhattan Psychiatric CenterNew York, NY, USA; ^3^Research Foundation for Mental Hygiene, Nathan Kline Institute for Psychiatric ResearchOrangeburg, NY, USA; ^4^Department of Psychology, New York UniversityNew York, NY, USA

**Keywords:** cognition, action video games, cognitive remediation, neurophysiology, cognitive enhancement

## Abstract

Action video game training may hold promise as a cognitive intervention with the potential to enhance daily functioning and remediate impairments, but this must be more thoroughly evaluated through evidence-based practices. We review current research on the effect of action video game training on visual attention and visuospatial processing, executive functions, and learning and memory. Focusing on studies that utilize strict experimental controls and synthesize behavioral and neurophysiological data, we examine whether there is sufficient evidence to support a causal relationship between action video game training and beneficial changes in cognition. Convergent lines of behavioral and neurophysiological evidence tentatively support the efficacy of training, but the magnitude and specificity of these effects remain obscure. Causal inference is thus far limited by a lack of standardized and well-controlled methodology. Considering future directions, we suggest stringent adherence to evidence-based practices and collaboration modeled after clinical trial networks. Finally, we recommend the exploration of more complex causal models, such as indirect causal relationships and interactions that may be masking true effects.

In September 2013, action video game Grand Theft Auto V broke all previous entertainment sales records by grossing $1 billion in just three days (Nayak, 2013). At present, video games yield $20 billion in annual sales and over 50% of Americans report owning a gaming console (Entertainment Software Association, 2013). Beyond entertainment value, video games are also extending into the domain of cognitive therapeutics: Lumosity, the industry leader in game-based cognitive enhancement, has amassed over 40 million users worldwide. These two game types, entertainment versus enhancement, appear qualitatively different. There are some researchers, however, who argue that video games designed for entertainment can facilitate meaningful improvements in cognitive function (Dye et al., 2009; Bavelier et al., 2012a). At present, these claims need to be more rigorously evaluated according to evidence-based practices before scientists endorse any potential therapeutic value.

Are cognitive benefits a direct consequence of video game training? In the only comprehensive meta-analysis on video games to date, Powers et al. (2013) report that playing video games yields a moderately positive effect on cognition. This effect is found in non-experimental studies, *d *= 0.46, 95% CI [0.39, 0.53], where expert video game players are compared to video game novices (hereafter referred to as “experts” and “novices”), as well as in true experiments, *d *= 0.45, 95% CI [0.35, 0.56], where participants train with a video game for a fixed period of time and are compared to their own initial performance or a control group. Yet non-experimental studies, by nature, preclude the possibility of strict causal inference, and a lack of rigorous and standardized methodology in the experimental training studies makes those findings vulnerable to possible confounds.

Powers et al. (2013) examined the specificity of cognitive effects in their moderator analysis. Unlike their primary analysis where they aggregated multiple test outcomes into a single summary effect for each study, their secondary analysis treated studies with multiple test outcomes as if each outcome originated from a separate and independent study. While they noted that this violates the assumption of independence, stating, “analysis at this level was required to test for the effects of most of the moderating variables” (p. 1059), treating multiple dependent outcomes as independent creates two major statistical confounds. One, it artificially inflates the cumulative sample size of the meta-analysis, and therefore overestimates the certainty of any findings, and two, it biases the weight of each study toward those with the greatest number of outcomes (see Borenstein et al., 2009). For example, in their examination of executive function their sample of 13 studies with a cumulative sample of 539, inflated to 89 studies with a cumulative sample of 3,721. Additionally, due to variability in the number of outcomes, some studies (e.g., Lee et al., 2012) were weighted 20 times more than others (e.g., Spence et al., 2009). Multiple dependent outcomes are a common problem in meta-analyses (Dunlap et al., 1996), and while there is no uniform consensus on how to account for them, there are a number of methods available to the researcher, the most common being multivariate methods (see Mavridis and Salanti, 2013). Thus, while Powers et al. (2013) have made a very worthwhile contribution to the field with their primary analysis, the findings reported in their moderator analyses should be interpreted with great caution.

To the extent that video games improve cognitive functioning, the critical next step is to determine what aspects of video games drive cognitive benefits, how this works, and what it targets in the brain. Additionally, different game types may have various effects on cognition or interact with specific domains. While evidence of neuroplastic change is necessary to establish causality, it is not sufficient. We must know what aspects of gameplay drive the change, as well as how and where it manifests in neural circuitry and observable behavior. Examining the literature, Boot et al. (2011, 2013; Boot and Simons, 2012 see also Kristjánsson, 2013) conclude that current research falls short of evidence-based practices. They lay out a series of methodological guidelines for future studies that include training paradigms that utilize randomization, active control groups, and better methods to account for placebo and practice effects They also suggest evaluating behavioral findings in conjunction with neurophysiological evidence in order to track cognitive changes alongside neural correlates.

The current review attempts to qualitatively address the issue of causality by adopting a strict focus on studies whose methodology provides the elements necessary for causal inference. We consider only experimental studies that include some form of training paradigm. We will not consider non-experimental, quasi-experimental studies, or correlational studies in this analysis. Within these studies we give the highest priority to those which include one or more of the following design elements: experimental control in the form of active and/or passive comparison groups, neuropsychological data to assess the transfer of training, and neurophysiological evidence to identify the structural or functional correlates of differences in cognitive performance. Unfortunately, there are few studies that include all of these design elements. Insufficient evidence exists to specify the exact “active ingredient” within video games; therefore we use a broad focus on games with an action component. For the purposes of this review we use a broad definition of the term action so as to not exclude games based on thematic or esthetic design elements. We summarize findings with respect to improvement in three cognitive areas: (1) visual attention and visuospatial processing, (2) executive functions, and (3) learning and memory. Lastly, we illustrate areas in need of further investigation and provide commentary for future directions.

## LEARNING AND MEMORY

Perhaps one of the more intriguing mechanisms of cognitive change relates to whether training on action video games can enhance one’s ability to efficiently learn novel tasks. The process of developing skills that facilitate learning in other contexts, referred to as “learning to learn” (Harlow, 1949), may underlie one’s capacity to benefit from training. Although within- and between-group differences may still play a role in learning to learn, Green and Bavelier (2008) argue that well-designed training procedures can facilitate cognitive enhancements that extend beyond specific experiments and conditions. These design principles include the use of shorter training periods, which may allow training effects to generalize more broadly (Karni and Sagi, 1993) and high variability in training strategies to facilitate learning (Schmidt and Bjork, 1992; Green and Bavelier, 2008). Improved learning may not be a specific target of training but rather the by-product of elaborate knowledge structures, complex learning algorithms, and more efficient allocation of attentional resources (Green et al., 2010; Bavelier et al., 2012b).

While attention and executive functions play a key role in learning to learn, it is less clear whether other aspects of cognition, such as memory, contribute toward this process. In particular, findings from training studies examining working memory have been inconsistent. Boot et al. (2008) report no significant between-group differences in any memory abilities post-training. While Basak et al. (2008) report no significant improvement in spatial memory, they do find a group-by-testing-session interaction in working memory. Given that Basak et al. (2008, 2011) did not utilize an active control group, these conflicting results may be due to expectation effects. Oei and Patterson (2013)Oei and Patterson (2013 find no post-training gains in spatial working memory, but do find improvements in working memory measured via a complex span task. Several recent reviews (Shipstead et al., 2012; Melby-Lervåg and Hulme, 2013) indicate that working memory training might improve performance on tasks similar to the training, but generalized skill transfer does not seem to occur. However, these reviews do not focus on action video games, which may provide a unique training experience.

Working memory is unique in that it requires not only maintaining information in short-term storage, but also places heavy demands on attention and continual response inhibition (Baddeley and Hitch, 1974). Behavioral findings of working memory gains may therefore be the result of improvements in other domains, namely attention and executive functioning. Although there are few neuroimaging studies that specifically address post-training working memory enhancement, preliminary data suggest that gains in executive functioning contribute significantly to behavioral findings. Basak et al. (2011) utilized structural MRI to compare brain volumes among older adults who underwent over 20 h of action video game training. The volume of the dorsolateral prefrontal cortex (DLPFC), an area critical to both working memory and executive functions, was correlated with improvements in game performance and with measurements of the rate of learning. This evidence suggests that enhancements in other cognitive domains may underlie working memory gains and provide a basis for understanding why other aspects of memory are unaffected by action video game training.

## EXECUTIVE FUNCTIONS

Several recent experimental studies support the idea that training increases various components of executive functioning (e.g., Basak et al., 2008; Green et al., 2012; Strobach et al., 2012). Anguera et al. (2013) reported that older adults without video game experience show enhanced cognitive control after training compared to both active and passive control groups. In terms of neurophysiology, action video game training appears to engage neural structures and circuits that mediate executive functions. EEG studies have shown associations between improved performance in executive function tasks and increases in both frontal alpha (Maclin et al., 2011; Mathewson et al., 2012) and midline frontal theta power (Anguera et al., 2013) after video game training.

In addition to post-training changes in brain function, there is preliminary evidence that training may also lead to structural changes. A recent study reports that compared to a passive control group, participants who trained on *Super Mario 64* for 30 min a day over a period of 2 months showed significant post-training group differences in gray matter volume in the right DLPFC, right hippocampus, and cerebellum (Kühn et al., 2014). While there was no time-by-group interaction for the hippocampus and cerebellum, the right DLPFC showed a significant interaction. The DLPFC is one of the most critical neuroanatomical areas for the executive functions and has been closely linked to the executive component of working memory (Goldman-Rakic, 1995) inhibitory control (Wager et al., 2005a) and shifting (Wager et al., 2005b). Although these results provide some support for structural brain changes post-training, the study lacked an active control group and therefore it is impossible to determine whether the changes resulted from the video game training itself or rather from engagement of any activity over the same period of time. As this is one of few studies to directly examine the effects of video game training on neurophysiology, we have included it in our analysis, despite this methodological flaw.

The relationship between action video game training and cognitive effects, however, may be more complex than a single cause and effect model. A structural imaging study by Erickson et al. (2010), found that pre-training volumes in both the ventral and dorsal striatum were associated with early-stage learning and skill acquisition in a game emphasizing cognitive flexibility, but that only dorsal volume was predictive of continued improvement. This suggests that while immediate learning and skill acquisition is likely related to reward processing and motivation (i.e., ventral striatum), progressive enhancements are a function of procedural learning and cognitive flexibility (i.e., dorsal striatum). Importantly, these findings also highlight the need to investigate how individual differences may be interacting with training to produce differential effects.

## VISUAL ATTENTION AND VISUOSPATIAL PROCESSING

The idea that action video games can influence cognition, and specifically attention, is long established (Greenfield, 1994; Greenfield et al., 1994a, b; Subrahmanyam and Greenfield, 1994). A landmark paper by Green and Bavelier (2003) paved the way for numerous behavioral studies concluding that video games modify visual attention and visuospatial processing. Visual attention and visuospatial processing have been grouped together in this review because of their close interaction and potential for bidirectional effects (Yeshurun and Carrasco, 1998, 1999; Carrasco et al., 2004). While these two processes may be independent at the neural level, this distinction is difficult to assess behaviorally. For example, post-training improvements in the continuous performance test (Riccio et al., 2002) or the useful field of vision task (Ball et al., 1993) may be the result of faster perceptual processing, more efficient prioritizing of visual information, or both. Although Green and Bavelier’s (2003) paper contained small-scale training studies that supported their conclusions, their subsequent work Green and Bavelier (2006a, b) focused on training novices as a way to identify whether superior attention in expert players was due to the games themselves or pre-existing differences. Novices showed enhancements in selective visual attention after training, suggesting that between-group differences alone could not account for observational findings. Similarly, Wu and Spence (2013) found that while novices initially exhibited poorer visual attention compared to experts, 10 h of training was sufficient to yield improvement. Other studies have produced similar findings (e.g., Feng et al., 2007); results, however, are not entirely consistent (e.g., Boot et al., 2008; Belchior et al., 2013).

Neurophysiological evidence also supports video game training’s ability to improve attention and visuospatial processing. Wu et al. (2012) found that participants who exhibited the most improvement on a behavioral measure of attention also showed increased evoked response potentials in late-stage visuospatial processing, compared to those with less attentional improvement and to control participants. These findings are interpreted as gains in the top-down allocation of attentional resources and improved distractor inhibition. Using functional neuroimaging, Prakash et al. (2012) reported that although both controls and training groups recruited attention control areas (such as the ventral medial prefrontal cortex) during task performance, subjects in the training group exhibited reduced activity post-training, suggesting enhanced top-down attentional control. Collectively, these results highlight the possibility that top-down control mediates the relationship between training and enhancements in attentional performance, and further suggest that the magnitude of improvement in attention and visuospatial processing may depend on an interaction between training and training strategy, and between training and individual differences. Future studies should extend upon this work by examining whether reduced activation post-training also correlates with improvements on tasks unrelated to the training paradigm, thus confirming the transferability of efficient neural network processing to non-training paradigms.

## DISCUSSION

The proliferation of video games as an entertainment medium provides an opportunity to better understand the plasticity of human cognition as a function of experience. Despite an incomplete understanding of these processes, the use of video games as a tool for cognitive enhancement has outpaced scientific evidence for its efficacy. We reviewed existing research on action video games and their training effects. Behavioral findings from training studies suggest improvements in attention, visuospatial processing, cognitive control and flexibility, but are inconclusive with respect to short-term memory. Enhancements in working memory do occur, although this could be secondary to improvements in attentional and executive resources. In many cases neurophysiological data bolster these behavioral findings through parallel evidence of neuroplastic change and elucidating potential underlying mechanisms related to enhanced cognitive function.

Boot et al. (2011) suggest adopting an experimental methodology, which mirrors that of clinical trials, including the use of active or placebo control groups, the improvement of reporting practices, and the reduction of demand characteristics. While this review provides support for these suggestions, it also highlights ways in which this approach can be advanced and extended. While most research has adopted a linear model, this precludes the possibility of indirect causality and assumes that training produces domain-specific enhancements equally across all subjects. More complex causal relations may be elucidated by mediation and moderation analyses, which future studies might explore. Neurophysiological evidence suggests that individual differences may underlie differential training effects (Erickson et al., 2010; Wu et al., 2012), indicating that training may not be equally beneficial for everyone. Future research should also explore individual characteristics as baseline predictors of treatment response.

Action video game studies should also strive to adopt methodologies that boost the signal of any training benefits while simultaneously reducing the noise of placebo effects. This includes adopting a randomized, double-blind, placebo-controlled clinical trial design (Pastore and Scheirer, 1974). Yet additional design considerations can also be utilized to maximize treatment effects. Increasing the signal of cognitive enhancement may be feasible through the adoption of training strategies that enhance complex skill acquisition. For example, regimens that emphasize variable training and sub-part training yield larger improvements over traditional repeated practice measures (Prakash et al., 2012). Clinical trials also benefit from pre-registration, a collaborative research network, and standardized methodology. This practice not only increases accountability, statistical power, and the consolidation of resources but also reduces the variability associated with disparate study designs.

Expectancy effects are another vital consideration in training studies, where active controls may not be enough for causal inference. Boot et al. (2013) survey participant expectations of the potential cognitive benefits of various games they had never played. They find significant differences in such expectations and conclude that unless these differences are accounted for, then causal inferences are potentially unreliable due to possible differential placebo effects. Future studies should standardize training tasks to minimize differences in expected benefits or include manipulation checks so that these differences can be statistically accounted for in analysis.

Additionally, researchers have not yet classified video games in a way that fully accounts for titles that blur genre lines. For example, MarioKart is a racing game in which players use various weapons and abilities to disrupt their opponents’ progress. Powers et al. (2013) categorized this as a non-action game, along with other sport and simulation games. Yet MarioKart appears to require many of the same cognitive and motoric demands of a traditional action game like Call of Duty. Even two games that are both readily accepted as first-person shooter games within the action genre, like Doom (1993) and *Call of Duty: Ghosts* (2013), have substantial variance in presentation of visual stimuli and in cognitive load. The multi-faceted nature of video games creates difficulties in appropriately categorizing game titles, and may be contributing to contradictory and confusing results in both the cognitive and neurophysiological studies of video games.

Lastly, it is crucial to consider the potential effects of individual differences. Future research should devote more attention to investigating what factors, if any, are more predictive of cognitive enhancement success. Affective domains such as motivation and reward sensitivity are underexplored, and, apart from a few studies (e.g., Erickson et al., 2010; Basak et al., 2011), baseline differences in brain structure and function may be worthy of greater investigation. Individual differences may be mediating and moderating the effects of video game training, and once identified these indirect effects may account for some of the observed heterogeneity in the literature.

Action video games deliver dynamic, multi-sensory stimulation that requires users to navigate tasks that are equally challenging and entertaining. This medium provides not only a unique tool for investigating human cognition and neuroplasticity, but also the means for potentially counteracting cognitive decline and remediating cognitive impairments. Yet such promise and opportunity must not supersede the need for rigorous and unbiased scientific evaluation. Action video game training may indeed lead to enhancements in attention, visuospatial processing, and executive functioning. However, the magnitude and specificity of these effects remain unclear. Future research should not only adopt methodologies based upon best practices from clinical trials, but also incorporate evidence from both behavioral and neurophysiological approaches.

## AUTHOR CONTRIBUTIONS

All authors participated in the literature review, drafted the initial manuscript sections with exception to the discussion, and provided critical revisions on the initial draft. Joseph Bisoglio, Timothy I. Michaels, and Joshua E. Mervis wrote the discussion and all authors contributed revisions to the final version of the manuscript, which all authors approved for submission.

## Conflict of Interest Statement

The authors declare that the research was conducted in the absence of any commercial or financial relationships that could be construed as a potential conflict of interest.
